# Relationships between employment status with self-perceived mental and physical health in Canada

**DOI:** 10.3934/publichealth.2024012

**Published:** 2024-02-29

**Authors:** Anson Kwok Choi Li, Behdin Nowrouzi-Kia

**Affiliations:** 1 Institute of Health Policy, Management and Evaluation, University of Toronto, 155 College Street, Suite 425, Toronto, ON, Canada M5T 3M6; 2 Department of Biology, University of Western Ontario, 1151 Richmond Street, London, ON, Canada N6A 3K7; 3 ReSTORE Lab, Department of Occupational Science and Occupational Therapy, Temerty Faculty of Medicine, University of Toronto, 500 University Avenue Toronto, ON, Canada M5G 1V7; 4 Krembil Research Institute-University Health Network, 60 Leonard Ave, Toronto, ON, Canada, M5T 0S8; 5 Centre for Research in Occupational Safety & Health, Laurentian University, 935 Ramsey Lake Rd, Sudbury, ON, Canada P3E 2C

**Keywords:** mental health, physical health, employment status

## Abstract

**Background:**

The annual cost of mental illnesses in Canada is estimated to be $50 billion. Research from other countries have suggested that employment status is associated with mental and physical health. Within the Canadian context, there is a dearth of research on the relationship between employment and mental health.

**Objective:**

To explore the relationships between age, gender, income, and employment status on mental and physical health.

**Methods:**

The 2021 Canadian Digital Health Survey dataset was used for this study. Data records, which included responses for the questions on age, gender, income, employment status, mental, and physical health, were used in the analysis. Ordinal logistics regression was applied to investigate the associations that may exist between mental and physical health with the various sociodemographic factors. Descriptive statistics were also provided for the data.

**Results:**

The total sample size included in the analysis was 10,630. When compared to respondents who had full-time employment, those who were unemployed were more likely to have lower self-perceived mental health (*OR*: 1.91; 95% *CI*: 1.55–2.34). Retired respondents were less likely to have worse mental health than respondents who were employed full-time (*OR*: 0.78; 95% *CI*: 0.68–0.90). Self-perceived physical health was more likely to be lower for those who were unemployed (*OR*: 1.74; 95% *CI*: 1.41–2.14) or retired (*OR*: 1.28; 95% *CI*: 1.12–1.48) when compared to respondents employed full-time. The likelihood of worsening mental and physical health was also found to be associated with age, gender, and income.

**Conclusion:**

Our findings support the evidence that different factors contribute to worsening mental and physical health. Full-time employment may confer some protective effects or attributes leading to an increased likelihood of having improved mental health compared to those who are unemployed. Understanding the complex relationships on how various factors impact mental health will help better inform policymakers, clinicians, and other stakeholders on how to allocate its limited resources.

## 1. Introduction

Approximately 1 in 5 Canadians experience mental illness annually [1]. The cost of mental illnesses in Canada is estimated to be $50 billion per year, including health care costs, lost productivity, and decreased quality of life [2]. Within the literature, there is evidence suggesting that a complex relationship between mental and physical health exists and that various factors can impact the relationship differently. Researchers found that higher systolic blood pressure was associated with positive mental health and wellbeing, while hypertension was associated with lower mental health and wellbeing [3]. Differences in the relationship between physical activity and mental health was found between sex in Canadian youths. Higher levels of physical activity was associated with lower depression symptoms and anxiety in males. However, in females, higher levels of physical activity was associated with elevated symptoms of anxiety [4]. In a study of older adults, it was found that physical activity had a positive influence on the relationship between resilience and mental health [5]. In addition, research on various socioeconomic factors, such as age, gender, and income found that they are associated with mental health [6],[7]. It is crucial to understand the various factors that could impact mental and physical health and which populations of Canadians are most impacted.

Previous studies have suggested a relationship exists between employment status and mental health. Research has found that unemployment was significantly associated with elevated risk of mental health problems, such as depression and suicide ideation, or overall poor mental health [8],[9]. An Australian study found evidence that employment had an indirect effect on the relationship between disability and the mental health of young adults [10]. The study found that employment status had a 0.91-point decline in mental health. Similarly, there is evidence in the literature supporting a relationship between employment status and physical health. A US study found that lower levels of employment from mid-to older adulthood in women were more likely to experience impairments in physical function [11].

A better understanding of the relationships between mental and physical health and various sociodemographic factors have helped to shape policy guidelines and recommendations. The World Health Organization's global guidelines on physical activity and sedentary behavior was established based on extensive research and review of the available evidence by a group of experts from various relevant scientific disciplines [12]. In Canada, a scoping review found that while no overarching policy or framework related to postsecondary mental health exists, the policy recommendations made at the national and provincial level were aligned in their priorities to shift the current treatment approach to mental health toward a more holistic, comprehensive, and collaborative one [13]. Research provides an evidence-based approach to shaping policy and establishing programs to address issues related to mental and physical health. For example, the Centre for Addiction and Mental Health (CAMH) launched an initiative in 2021 to help young people facing barriers to enter the job market due to mental health challenges [14]. Further research into the relationship between mental and physical health with sociodemographic factors such as employment status will help to provide additional evidence and support for the establishment and funding of these types of programs in Canada.

To our knowledge, the research studies that have investigated the relationships between mental health with employment status in Canada have mainly focused on specific subsets of the Canadian population or were comparisons of sociodemographic factors within a particular occupation or employment status [15]–[20]. While the importance of these research studies cannot be understated, there is a dearth of research into the relationships between employment status and mental and physical health at that national level that covers a broad representative sample of the overall Canadian population. Addressing this gap in knowledge in Canada will inform policymakers, researchers, and other stakeholders on guideline recommendations and identifying which populations of Canadians, based on sociodemographic factors, would benefit the most from targeted programs to improve mental and physical health.

Our objective of this cross-sectional study was to analyze data regarding self-perceived physical and mental health from the 2021 Canadian Digital Health Survey across a wide population of Canadians (based on age, gender, income, and employment status).

## 2. Methods

### 2.1. Recruitment and data collection

Data was obtained from the 2021 Canadian Digital Health Survey, a cross-sectional survey of 12,052 Canadians over 16 years of age. The survey was administered and data collected between July 14 and August 6, 2021. It was commissioned by Canada Health Infoway and conducted by Leger via computer-assisted web interviewing technology.

Survey participants were selected from the Leger Opinion panel, a group of 500,000 representative panelists from various regions across Canada. The panelists were randomly selected, and participants from hard-to-reach target groups were added to the panel through targeted recruitment campaigns [21]. Survey respondents resided in one of the following provinces or territories:

1 - British Columbia/Territories.

2 - Canadian Prairies.

3 - Ontario.

4 - Quebec.

5 - Atlantic Canada.

The 2021 Canadian Digital Health Survey is a publicly-available dataset that can be accessed via: https://borealisdata.ca/. Research ethics board approval was not required for this study since the data was made publicly-available by Canada Health Infoway.

### 2.2. Dependent variable measures

The two dependent variables included the respondents' self-reported physical and mental health. The following questions were specifically used:

Q12: In general, how would you rate your overall physical health?

Q13: In general, how would you rate your overall mental health?

For both questions, respondents selected one response from the following 5-point Likert Scale:

1 - Excellent.

2 - Very Good.

3 - Good.

4 - Fair.

5 - Poor.

Data points were not included in the analyses if a respondent answered “Prefer not to say” to either of the two questions.

### 2.3. Independent variable

The four independent variables and its options used in this study included:

Age: (1) 16–24 years; (2) 25–34; (3) 35–54; (4) 55–64; and (5) 65+.

Gender: (1) Man; (2) woman; and (3) other including non-binary, two-spirit, transgender, prefer not to answer and other gender identities.

Household income: (1) < $24,999; (2) $25,000–$49,999; (3) $50,000–$79,999; (4) $80,000–$99,000; (5) $100,000–$149,999; (6) $150,000–$249,999; (7) $250,000+; and (99) prefer not to answer.

Employment status: (1) Working full-time; (2) working part-time; (3) homemaker, no outside employment; (4) unemployed; (5) retired; (6) disabled; (7) student; (96) other; and (99) prefer not to answer.

Age was calculated as the difference between a respondent's birth year and the survey date.

Data points were not included in the analyses if a respondent answered “Prefer not to answer” for the question regarding household income. In addition, data points were not included in the analyses if a respondent answered either “Other” or “Prefer not to answer” for the question regarding employment status.

### 2.4. Statistical analysis

Ordinal logistic regression models were used to investigate the associations between the dependent and independent variables. The dependent variables were transformed into ordinal categories and ranked on an ordinal scale from highest (1) to lowest (5), reflecting the ordered nature of the response options. The independent variables were coded as factors within the ordinal logistic regression model.

The reference group selected for employment status was “Working full-time” as it was the normative category for this study which the other employment statuses were assessed against. The reference groups selected for age and household income were “16–24 years” and “<$24,999”, respectively, as it represented the lowest response options available for both categories. The reference group for gender was the response option “Man” (male).

The statistical analyses were conducted using RStudio Version 2023.9.1.494 (Windows 11 Pro Operating System) [22]. Ordinal logistic regression models were fitted to explore the relationships between the dependent and independent variables. Its aim was to assess the relationship of age, gender, income, and employment status with the ordered response categories.

## 3. Results

### 3.1. Descriptive statistics

**Figure 1.**
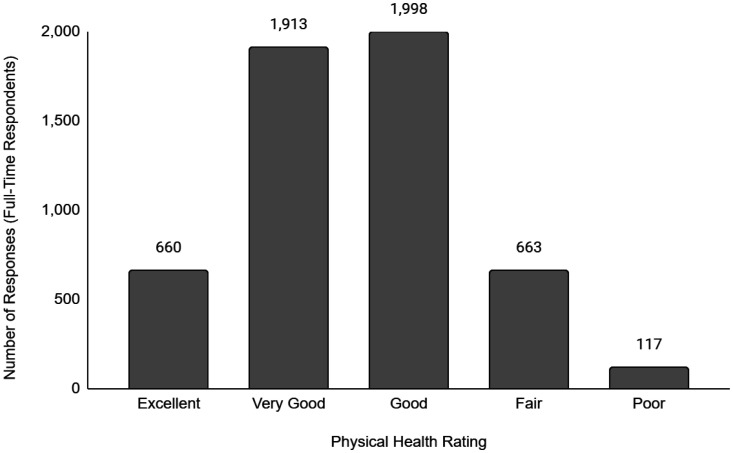
Frequency of full-time respondents' ratings on their overall physical health.

Descriptive data of the respondents' employment and sociodemographic characteristics are shown in Table 1. Breakdown of the provinces/territories where the respondents reside are provided in Table 2. The frequency of responses for Q12 - Physical Health and Q13 - Mental Health stratified by employment status are provided in Figures 1 to Figure 14. The frequency of responses for age, household income, and gender are provided as Supplementary Figures.

**Table 1.** Descriptive statistics of respondents based on socioeconomic and demographic characteristics.

**Factor**	**Category**	**Sample Size (Male)**	**Sample Size (Female)**	**Sample Size (Other)**
Age	16–24 25–34 35–54 55–64 65+	309 (30.8%) 735 (43.5%) 972 (50.2%) 1090 (50.8%) 2080 (54%)	660 (65.8%) 936 (55.4%) 944 (48.8%) 1043 (48.6%) 1759 (45.6%)	34 (3.4%) 20 (1.2%) 20 (1.0%) 13 (0.6%) 15 (0.4%)
Income	< $24,999 $25,000–$49,999 $50,000–$79,999 $80,000–$99,000 $100,000–$149,999 $150,000–$249,999 $250,000+	397 (38.5%) 862 (43.6%) 1186 (48.2%) 857 (51.2%) 1143 (53.7%) 592 (53.8%) 149 (58.7%)	606 (58.8%) 1094 (55.3%) 1258 (51.1%) 807 (48.2%) 972 (45.6%) 503 (45.7%) 102 (40.2%)	27 (2.6%) 23 (1.2%) 19 (0.8%) 9 (0.5%) 15 (0.7%) 6 (0.5%) 3 (1.2%)
Employment status	Working full-time Working part-time Homemaker, no outside employment Unemployed Retired Disabled Student	2938 (54.9%) 440 (38.6%) 51 (13.2%) 236 (47.8%) 1207 (52.5%) 129 (39.7%) 185 (29.1%)	2372 (44.3%) 689 (60.4%) 330 (85.7%) 249 (50.4%) 1086 (47.2%) 186 (57.2%) 430 (67.6%)	41 (0.8%) 11 (1.0%) 4 (1.0%) 9 (1.8%) 6 (0.3%) 10 (3.1%) 21 (3.3%)

**Table 2.** Breakdown of respondents based on province/territory.

**Province/Territory**	**Number of Respondents (%)**
British Columbia/Territories Canadian Prairies Ontario Quebec Atlantic Canada	1170 (11.0%) 2001 (18.8%) 4249 (40.0%) 2528 (23.8%) 682 (6.4%)

**Figure 2.**
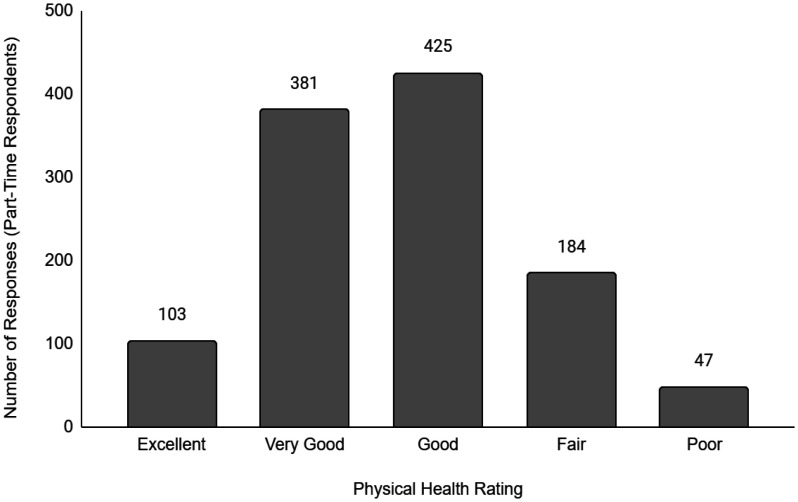
Frequency of part-time respondents' ratings on their overall physical health.

**Figure 3.**
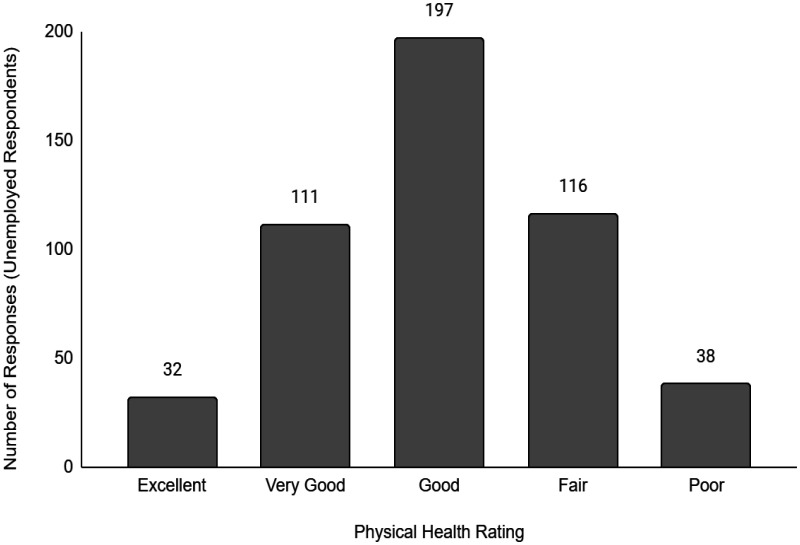
Frequency of unemployed respondents' ratings on their overall physical health.

**Figure 4.**
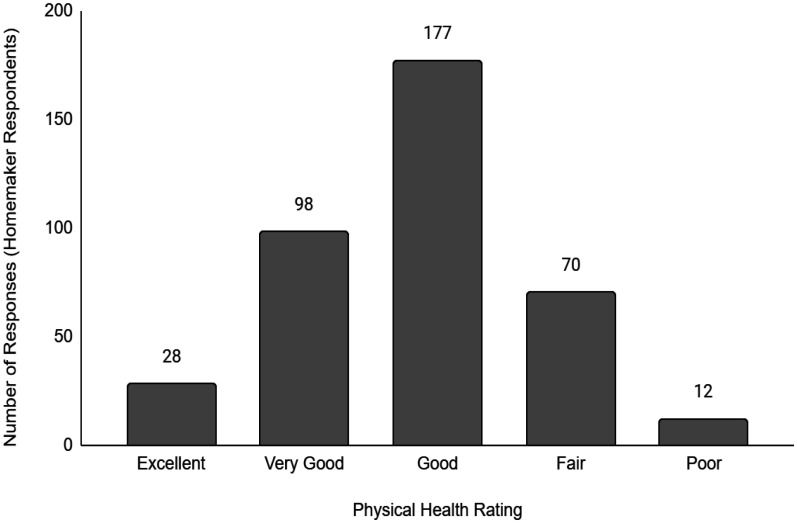
Frequency of homemaker respondents' ratings on their overall physical health.

**Figure 5.**
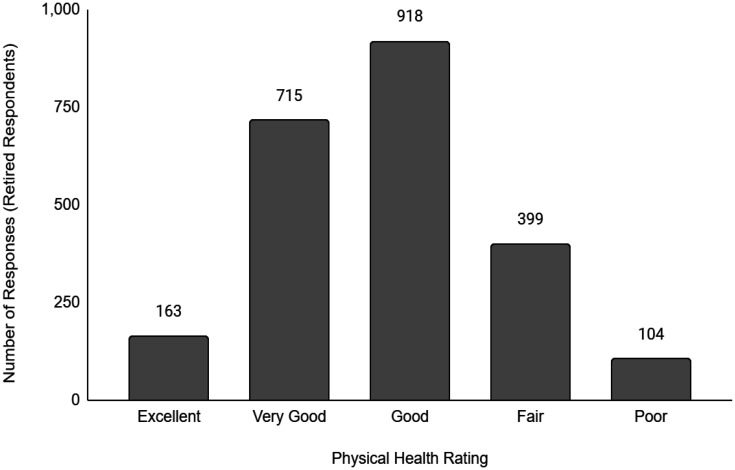
Frequency of retired respondents' ratings on their overall physical health.

**Figure 6.**
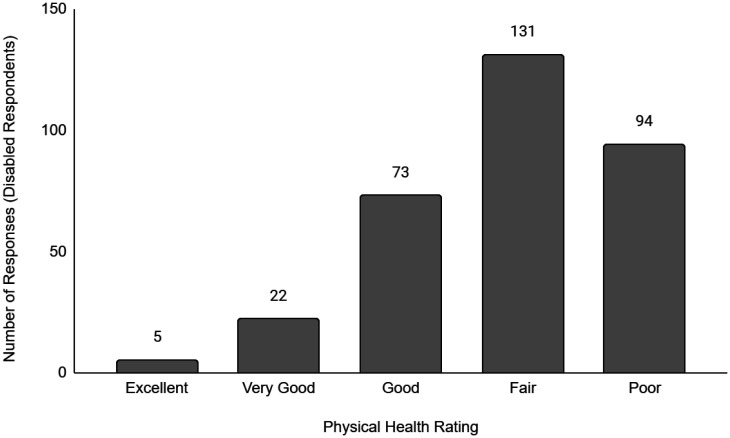
Frequency of disabled respondents' ratings on their overall physical health.

**Figure 7.**
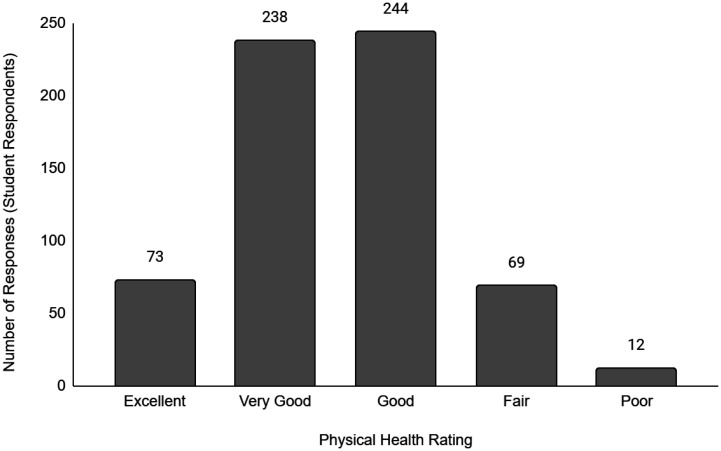
Frequency of student respondents' ratings on their overall physical health.

**Figure 8.**
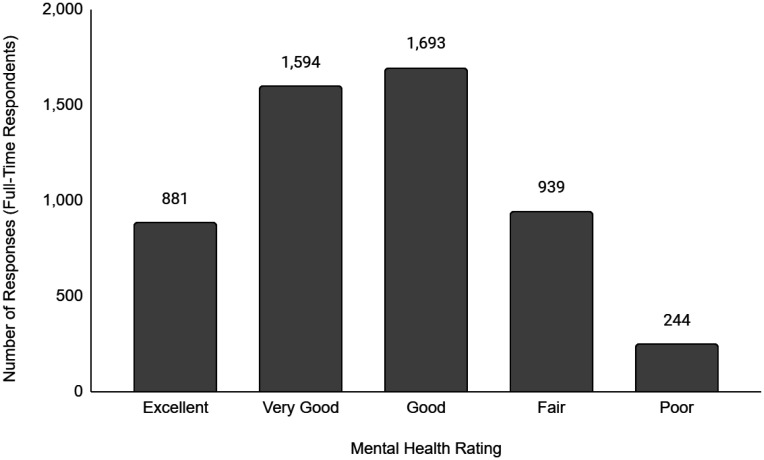
Frequency of full-time respondents' ratings on their overall mental health.

**Figure 9.**
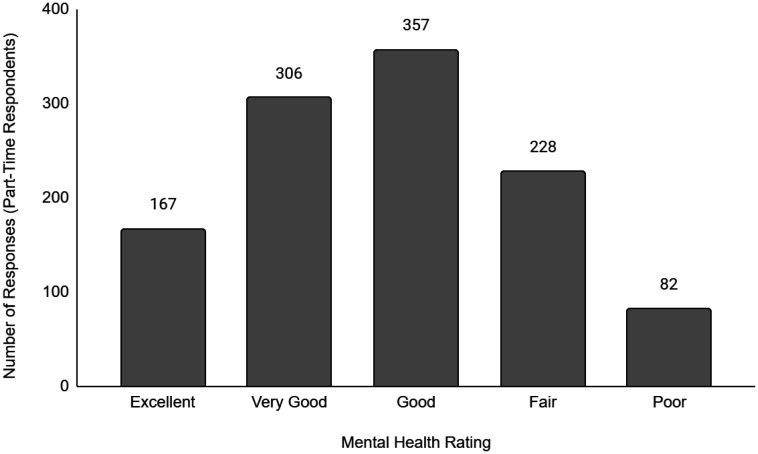
Frequency of part-time respondents' ratings on their overall mental health.

**Figure 10.**
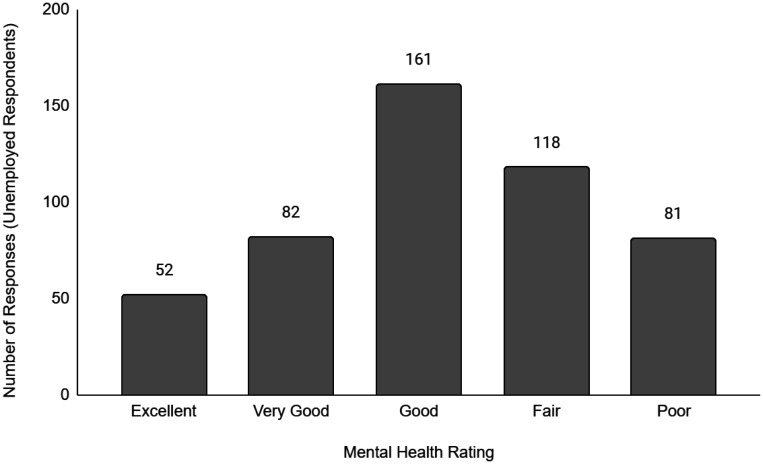
Frequency of unemployed respondents' ratings on their overall mental health.

**Figure 11.**
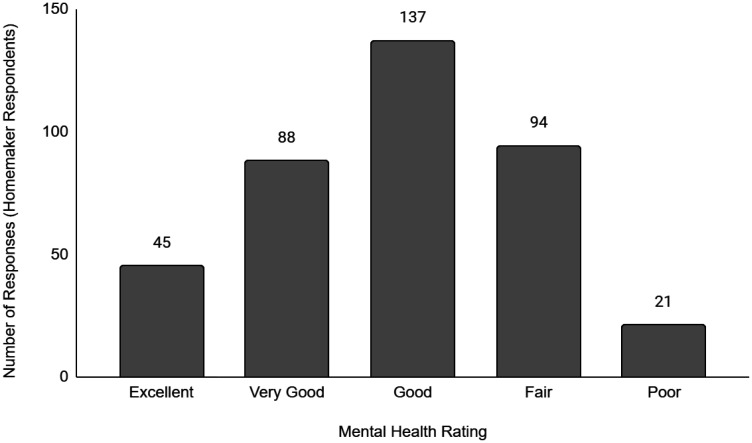
Frequency of homemaker respondents' ratings on their overall mental health.

**Figure 12.**
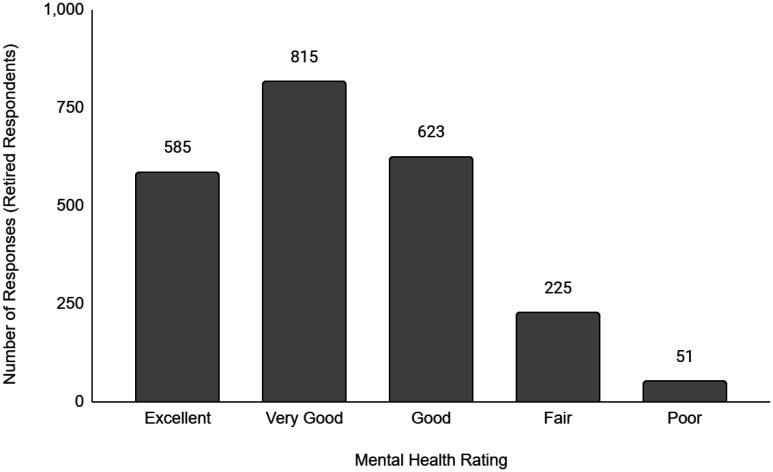
Frequency of retired respondents' ratings on their overall mental health.

**Figure 13.**
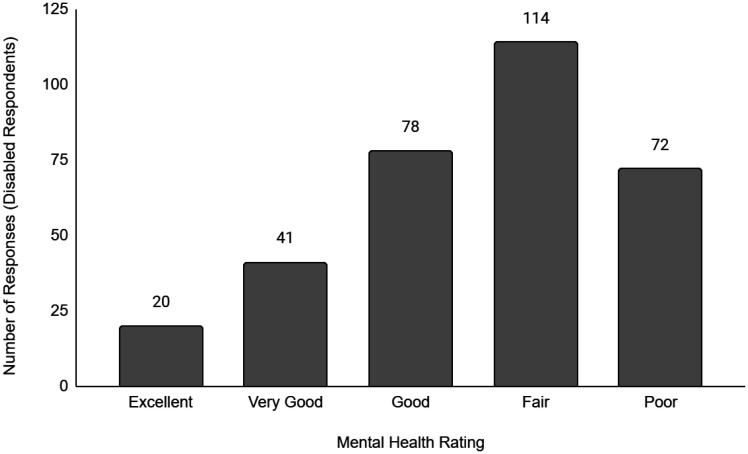
Frequency of disabled respondents' ratings on their overall mental health.

**Figure 14.**
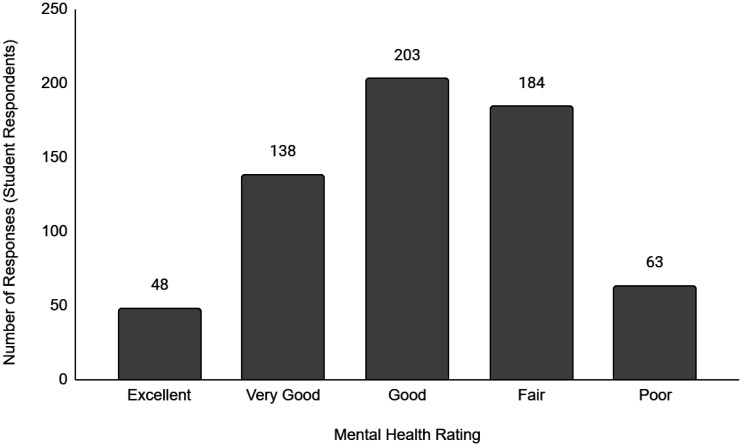
Frequency of student respondents' ratings on their overall mental health.

### 3.2. Ordinal logistic regression analysis

Several significant correlations were found between the employment status, sociodemographic factors, and the dependent variables (Tables 3 and 4).

### 3.3. Sociodemographic factors

Self-perceived physical health was more likely to be worse with increasing age. Respondents in the 65+ age group were 1.61 times more likely to have worse physical health than respondents in the 16–24 age group (*OR* = 1.61, *p* < 0.001). Conversely, self-perceived mental health was more likely to be higher with increasing age, suggesting that younger respondents rated having worse mental health than older respondents. Respondents in the 65+ age group were 66% less likely to have worse mental health than respondents in the 16–24 age group (*OR* = 0.34, *p* < 0.001).

Both self-perceived physical and mental health were significantly more likely to be worse in females and those who identified as ‘Other’ compared to males.

Increasing levels of income appeared to have a positive effect on both self-perceived physical and mental health. For each increasing income group, the odds ratio decreased to a larger degree than the preceding income group. A trend exists such that with each increasing income group, the likelihood of having worse levels of physical and mental health was significantly lower than the lowest income group. For respondents in the $250,000+ income group, the odds of having worsening levels of physical and mental health were 71% (*OR* = 0.29, *p* < 0.001) and 61% (*OR* = 0.39, *p* < 0.001) lower, respectively, than respondents in the <$24,999 income group. In comparison, respondents in the $25,000–$49,999 income group were only 31% (*OR* = 0.69, *p* < 0.001) and 19% (*OR* = 0.81, *p* < 0.001) less likely to report having worse levels of physical and mental health, respectively, than respondents in the <$24,999 income group.

### 3.4. Employment status

Compared to working full-time, there was no significant difference in the likelihood of having worse physical or mental health for students or respondents working part-time. However, respondents who were unemployed were 1.74 times and 1.91 times more likely to have worse physical (*OR* = 1.74, *p* < 0.001) and mental health (*OR* = 1.91, *p* < 0.001) levels than compared to respondents working full-time. For disabled respondents, the likelihood of having worse physical and mental health was more pronounced at 8.10 times (*OR* = 8.10, *p* < 0.001) and 4.35 times (*OR* = 4.35, *p* < 0.001), respectively, greater than respondents working full time. While retired respondents were 1.28 times (*OR* = 1.28, *p* < 0.001) more likely to have worse physical health than full-time workers, they were 22% more likely (*OR* = 0.78, *p* < 0.001) to have better mental health than full-time workers.

**Table 3.** Likelihood of self-perceived physical health being rated lower than the reference group.

**Dependent Variable**	**Factor**	**Categories**	***Odds Ratio***	**95% *CI***	***p*-value**
Q12: In general, how would you rate your overall physical health?	Age	16–24 25–34 35–54 55–64 65+	1.00 (Ref) 1.11 1.41 1.79 1.61	N/A (0.92, 1.34) (1.16, 1.71) (1.47, 2.17) (1.32, 1.97)	N/A 0.286 0.001 <0.001 <0.001
	Gender	Male Female Other	1.00 (Ref) 1.09 1.66	N/A (1.00, 1.18) (1.09, 2.52)	N/A 0.047 0.018
	Income	< $24,999 $25,000–$49,999 $50,000–$79,999 $80,000–$99,000 $100,000–$149,999 $150,000–$249,999 $250,000+	1.00 (Ref) 0.69 0.60 0.54 0.44 0.39 0.29	N/A (0.59, 0.82) (0.51, 0.71) (0.45, 0.64) (0.37, 0.52) (0.32, 0.47) (0.21, 0.39)	N/A <0.001 <0.001 <0.001 <0.001 <0.001 <0.001
	Employment Status	Working full-time Working part-time Homemaker, no outside employment Unemployed Retired Disabled Student	1.00 (Ref) 1.15 1.49 1.74 1.28 8.10 1.06	N/A (1.00, 1.32) (1.20, 1.85) (1.41, 2.14) (1.12, 1.48) (6.27, 10.48) (0.85, 1.32)	N/A 0.053 <0.001 <0.001 <0.001 <0.001 0.592

**Table 4.** Likelihood of self-perceived mental health being rated lower than the reference group.

**Dependent Variable**	**Factor**	**Categories**	***Odds Ratio***	**95% *CI***	***p*-value**
Q13: In general, how would you rate your overall mental health?	Age	16–24 25–34 35–54 55–64 65+	1.00 (Ref) 0.65 0.56 0.54 0.34	N/A (0.54, 0.78) (0.46, 0.68) (0.44, 0.65) (0.28, 0.41)	N/A <0.001 <0.001 <0.001 <0.001
	Gender	Male Female Other	1.00 (Ref) 1.45 2.81	N/A (1.33, 1.57) (1.77, 4.48)	N/A <0.001 <0.001
	Income	< $24,999 $25,000–$49,999 $50,000–$79,999 $80,000–$99,000 $100,000–$149,999 $150,000–$249,999 $250,000+	1.00 (Ref) 0.81 0.72 0.60 0.57 0.52 0.39	N/A (0.68, 0.95) (0.61, 0.85) (0.51, 0.71) (0.48, 0.68) (0.43, 0.64) (0.29, 0.53)	N/A <0.001 <0.001 <0.001 <0.001 <0.001 <0.001
	Employment Status	Working full-time Working part-time Homemaker, no outside employment Unemployed Retired Disabled Student	1.00 (Ref) 1.07 1.32 1.91 0.78 4.35 1.11	N/A (0.93, 1.24) (1.06, 1.64) (1.55, 2.34) (0.68, 0.90) (3.37, 5.61) (0.89, 1.37)	N/A 0.314 0.013 <0.001 <0.001 <0.001 0.348

## 4. Discussion

Our findings demonstrate that the employment status of Canadians are associated with both mental and physical health. It also supports the evidence in the literature of the relationships between sociodemographic factors, such as age, gender, and income with mental and physical health.

### 4.1. Employment status

The mental health of respondents who were unemployed, disabled, or homemakers were significantly more likely to be worse than compared to respondents working full-time. While the causal relationship could not be determined from these findings given the cross-sectional nature of this study, there is evidence in the literature that employment has a positive impact on mental health [23]–[25]. Employment can improve mental health and it can lead to increased self-reliance, self-confidence, and other positive health outcomes. Recently, there have been calls for changes in mental health treatment to include supported employment. Our findings suggest that employment may confer benefits or improvements to one's mental health compared to unemployment. One study proposed that this difference in mental health between employed and unemployed individuals is due to latent functions, including the lack of time structure, social contact, collective purpose, status, and activity. Interestingly, a meta-analysis found that the latent functions related to employment, which were significant predictors of mental health, did not differ between homemakers and employed people [26]. We found that homemakers were significantly more likely to have worse mental health than employed respondents. Overall, the findings from this study suggests that some protective effects or attributes about employment may be missing in unemployment and the homemakers, resulting in an increased likelihood of having worse mental health in the latter groups.

The results point to a significant association between unemployment and decreased mental health, but the interplay between the two is not readily known. A positive feedback loop may exist, where ongoing unemployment and worsening mental health continue to further exacerbate each other. Further funding for programs and initiatives in Canada, such as the one CAMH recently established to help those with mental illnesses find employment, will help to address and reduce this potential positive feedback between unemployment and mental health [14].

The literature on the impact that retirement has on mental health is mixed, and it may also be country-dependent. A Chinese study found that retirement significantly reduced depression and had a positive impact on their mental health [27]. A study of women in the US found differences in their mental health between forced and voluntary retirement [28]. Within Canada, a study found that retirement did not have a significant impact on depression and suggested that the direction of the effect may point to increased depression symptoms and worsening of mental health [29]. Our results support the finding that retirement improves overall mental health. However, given the nuanced nature of this topic, further research is needed to understand the impact of retirement on mental health. In addition, the Canadian Digital Health Survey does not distinguish between sub-populations of retirees, for example, voluntary vs involuntary retirement, early vs delayed retirement, and complete vs partial retirement. This information will be important to Canadian policymakers in understanding whether retirees are likely to increase or decrease mental health care expenditure.

The physical health of respondents generally followed the same trend as mental health when compared against employment status, with the exception of retired respondents. For the retired respondents, their physical health was more likely to be perceived as worse than those of full-time workers. The literature on retirement and its impact on physical health is sparse and mixed. One study found that while early retirement resulted in the worsening of health outcomes, delayed retirement also offered no health benefits [30]. Another study found that retirement may be beneficial to physical health for retirees with poor health and limited resources. However, retirement may not confer any health benefits to retirees who are wealthy and healthy [31]. The relationship between retirement and physical health may be more nuanced with variations existing between sub-populations of retirees. The research on the association between physical health and employment status is limited in the literature and should be an area of future research.

### 4.2. Socioeconomic factors

We found a significant trend between income and both mental and physical health. As income increased, the likelihood of worsening mental and physical health was significantly decreased. These findings support the wealth of research that has shown the relationship between income and health [32]–[34], as lower levels of income are associated with poorer mental and general health. Addressing income inequality will not only help to improve mental and physical health in Canadians, but research has shown that it has a significant role in determining other health outcomes [35]. A study using the Canadian Community Health Survey (CCHS) 2013–2014 dataset found evidence of inequities in access to mental health services based on income and suggested that there may be less social support for lower-income people living with mental health issues [36]. Using the 2021 Canadian Digital Health Survey dataset, our findings of decreased mental health with lower income levels suggests that income-based mental health inequities may continue to persist today in the Canadian population.

Increasing age had opposite effects on mental and physical health. Physical health appeared to be worse with age, however, the likelihood of worsening mental health decreased with age. The results suggest that younger Canadians have worse levels of mental health compared to older Canadians. Significant gender differences in mental and physical health were found. Females and respondents identifying as ‘Other’ which included non-binary, two-spirit, transgender, prefer not to answer, and other gender identities were significantly more likely to have worse mental and physical health compared to male respondents. These findings support the body of research that has found that gender differences exist for mental health. One study found that women are more likely to experience mental disorders, such as depression and anxiety, than compared to men and the relationship was consistent across younger and older age groups [37]. Another study found that transgender adults had higher odds of exhibiting frequent mental distress and of reporting a lifetime depression diagnosis, compared to cisgendered adults [38].

## 5. Limitation

Self-reporting bias was a limitation of this study as data from the Canadian Digital Health Survey, a self-reported survey, was used. Given the general nature of the questions in the survey, the interpretation of overall mental and physical health will differ between respondents. However, due to the large and varied sample size, the results provide relevant insight into the relationships between mental and physical health, employment status, and other socioeconomic factors. In addition, we were not able to explain the causal relationship between employment status and mental and physical health since this was a cross-sectional study.

## 6. Conclusion

Our findings contribute to the body of literature on social determinants of health. It provides further data and evidence of the multifactorial nature of mental and physical health within the Canadian context.

Overall, the lack of employment, including unemployed people and homemakers with no outside employment, is associated with decreased mental and physical health compared to people with full-time employment. The relationship between retirement and mental health may be highly nuanced. Further research should investigate various sub-populations of retirees to attain a fulsome picture of its relationship. Additional research into the causal relationships between employment and unemployment with mental health should be investigated. Our findings of the relationship between unemployment and mental health may point to a positive feedback loop, where the inability to find work and worsening mental health continue to exacerbate each other.

While we focused on sociodemographic factors, we acknowledge that there are a multitude of diverse factors that impact mental health that were not explored here. For example, previous studies have found that various factors, such as physical activity, sleep quality, racial differences, religion, and provision of financial incentives, contribute to mental and physical health in Canadians [39]–[42]. Addressing mental health issues in Canada has become a national priority within healthcare, however funding has not kept up and would thus limit how it is allocated [43]. As a result, it is important to understand the sociodemographic profile of Canadians most associated with decreased mental health. Given the high direct and indirect cost of mental health to the Canadian healthcare system, understanding the complex relationships that impact mental health will help better inform policymakers, clinicians, and other stakeholders on how to allocate its resources.

## Use of AI tools declaration

The authors declare no Artificial Intelligence (AI) tools have been used in the creation of this article.


