# Supplementary material for: Relationships between employment status with self-perceived mental and physical health in Canada
Source: AIMS Public Health. 2024 Feb 29;11(1):236–57. doi: 10.3934/publichealth.2024012 (PMC11007413; doi:10.3934/publichealth.2024012)
Supplement: Supplementary file 1 [file publichealth-11-01-012-s001.pdf]

AIMS Public Health

AIMS Public Health, 11(1): 236–257.
DOI: 10.3934/publichealth.2024012
Received: 22 January 2024
Revised: 22 February 2024
Accepted: 27 February 2024
Published: 29 February 2024

http://www.aimspress.com/journal/aimsph

Brief report

Relationships between employment status with self-perceived mental and physical health in Canada

Anson Kwok Choi Li[1,2,3] **and Behdin Nowrouzi-Kia**[3,4,5,*]

[1] Institute of Health Policy, Management and Evaluation, University of Toronto, 155 College Street, Suite 425, Toronto, ON, Canada M5T 3M6
[2] Department of Biology, University of Western Ontario, 1151 Richmond Street, London, ON, Canada N6A 3K7
[3] ReSTORE Lab, Department of Occupational Science and Occupational Therapy, Temerty Faculty of Medicine, University of Toronto, 500 University Avenue Toronto, ON, Canada M5G 1V7
[4] Krembil Research Institute-University Health Network, 60 Leonard Ave, Toronto, ON, Canada, M5T 0S8
[5] Centre for Research in Occupational Safety & Health, Laurentian University, 935 Ramsey Lake Rd, Sudbury, ON, Canada P3E 2C

* **Correspondence**: E-mail: behdin.nowrouzi.kia@utoronto.ca.

Supplementary

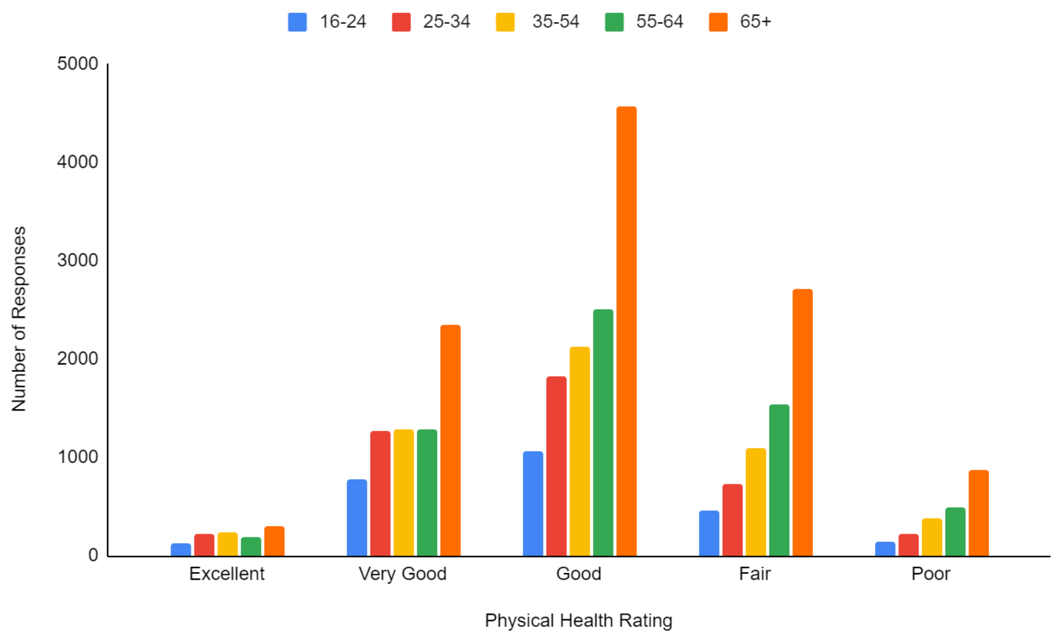

Figure S1. Frequency of respondents' ratings on their overall physical health based on age.

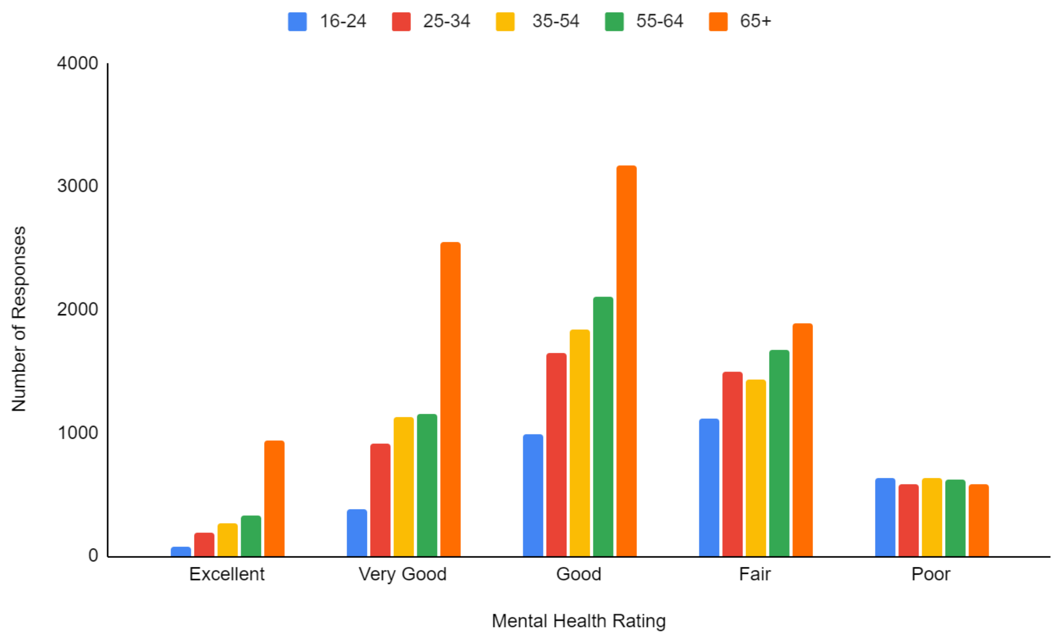

Figure S2. Frequency of respondents' ratings on their overall mental health based on age.

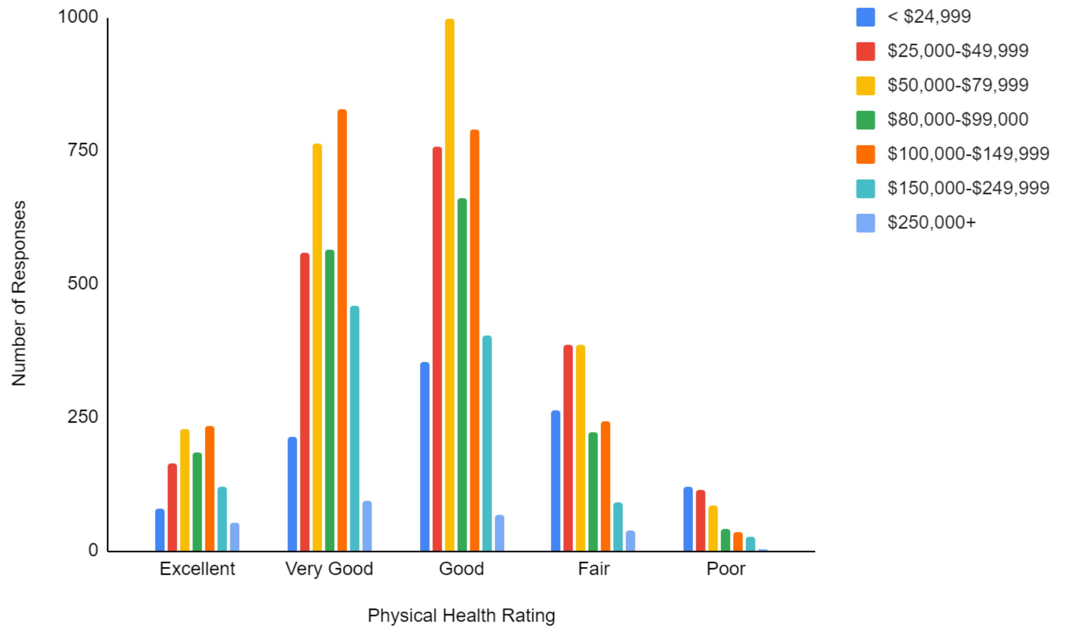

Figure S3. Frequency of respondents' ratings on their overall physical health based on household income.

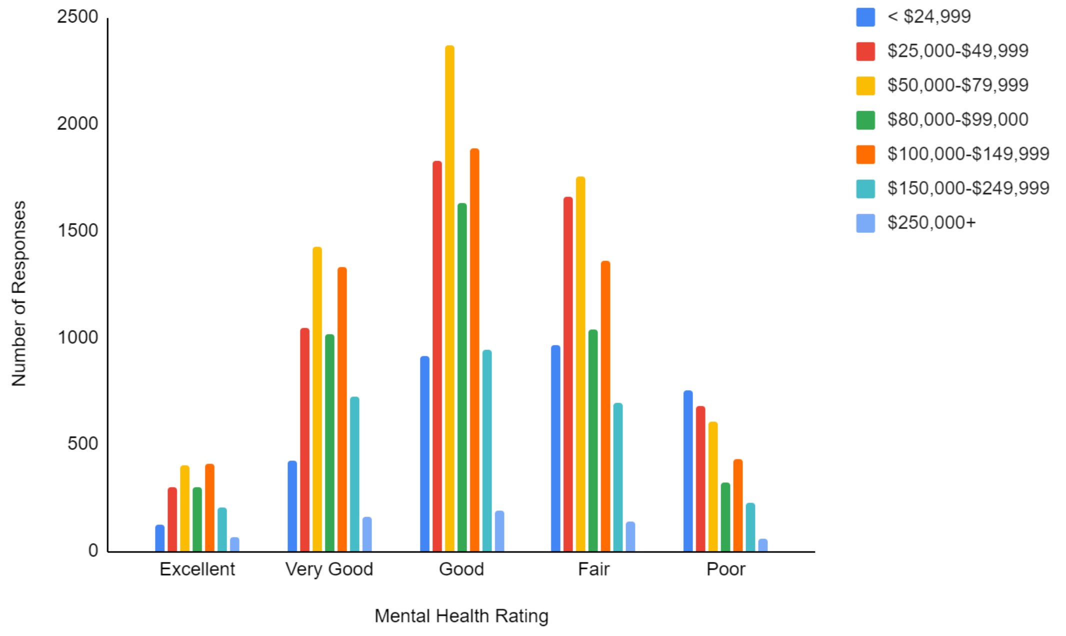

Figure S4. Frequency of respondents' ratings on their overall mental health based on household income.

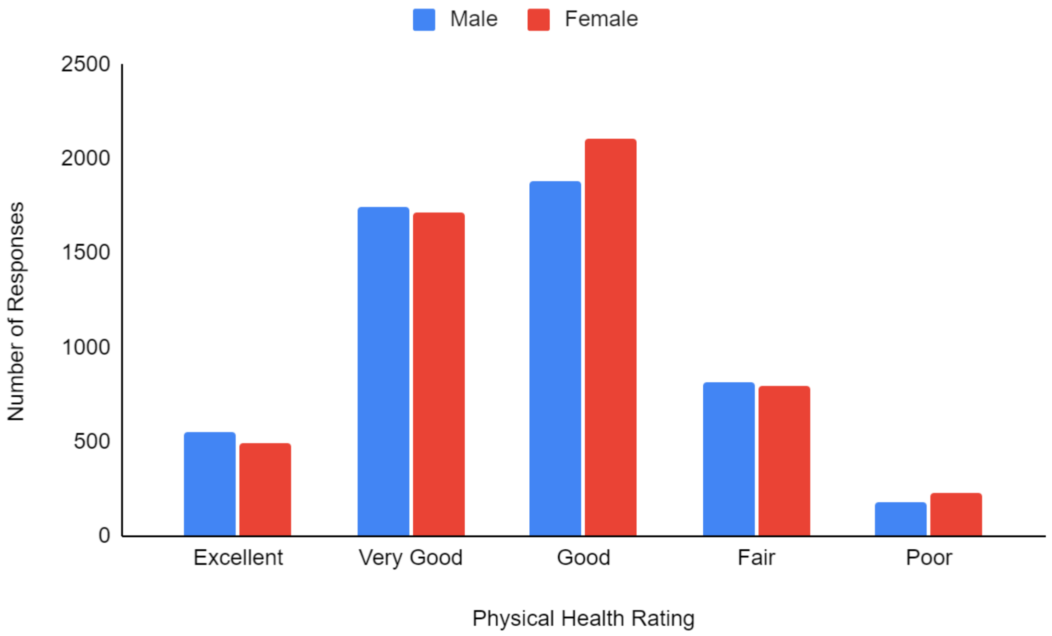

Figure S5. Frequency of male and female respondents' ratings on their overall physical health.

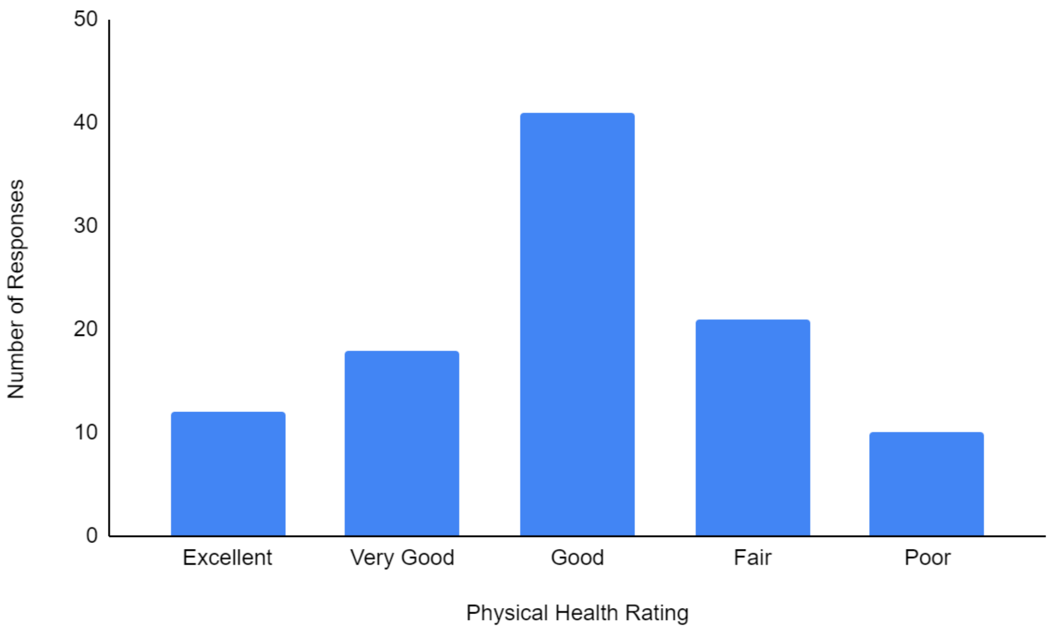

Figure S6. Frequency of 'Other' respondents' ratings on their overall physical health.

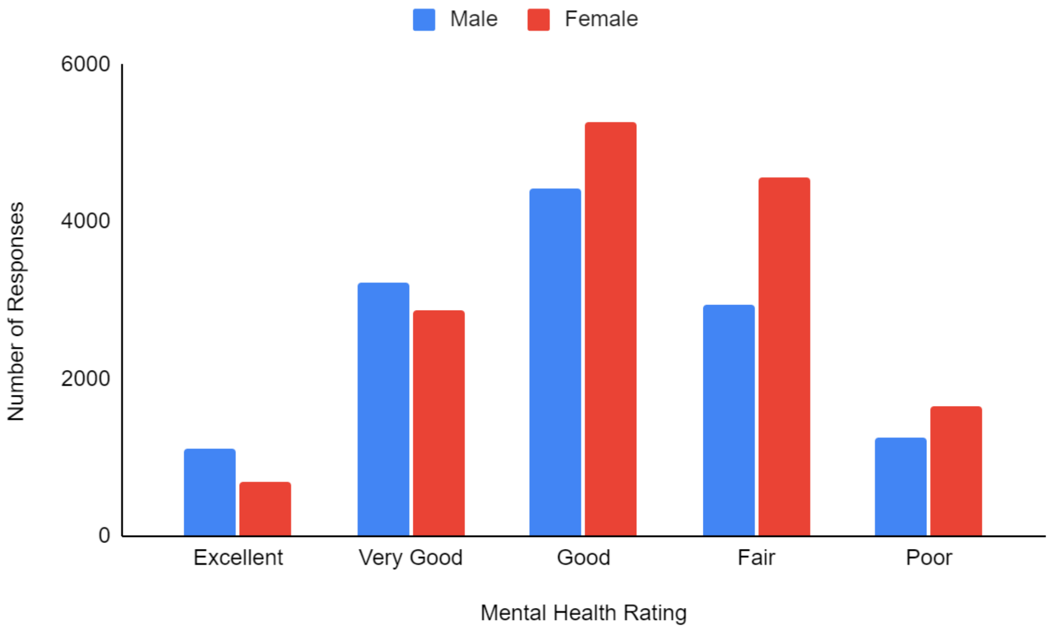

Figure S7. Frequency of male and female respondents' ratings on their overall mental health.

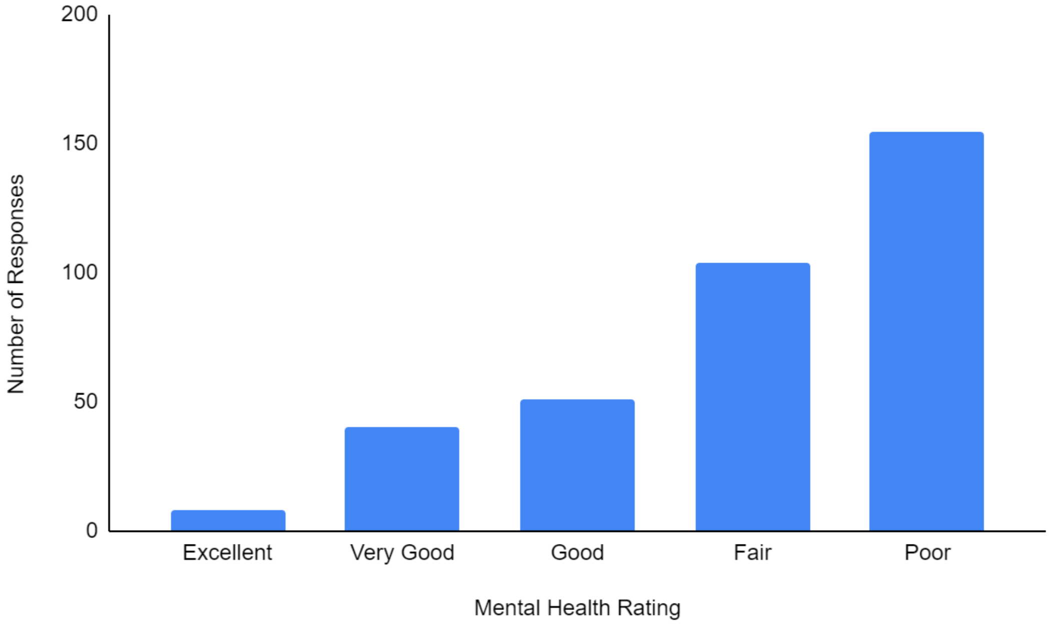

Figure S8. Frequency of 'Other' respondents' ratings on their overall mental health.

AIMS Press

© 2024 the Author(s), licensee AIMS Press. This is an open access article distributed under the terms of the Creative Commons Attribution License (http://creativecommons.org/licenses/by/4.0)
